# MicroRNA‐9 exerts antitumor effects on hepatocellular carcinoma progression by targeting HMGA2

**DOI:** 10.1002/2211-5463.12716

**Published:** 2019-09-20

**Authors:** Xiangang Xu, Haibo Zou, Lanyun Luo, Xiankui Wang, Guan Wang

**Affiliations:** ^1^ Department of Hepatobiliary Surgery Guizhou Provincial People's Hospital Guiyang China; ^2^ Department of Hepatobiliary Surgery Sichuan Academy of Medical Sciences and Sichuan Provincial People's Hospital Chengdu China

**Keywords:** antitumor, hepatocellular carcinoma, high mobility group AT‐hook 2, miR‐9, miRNA

## Abstract

Accumulating evidence has demonstrated that the aberrant expression of microRNAs (miRs or miRNAs) may contribute to the initiation and progression of various types of human cancer and may also constitute biomarkers for cancer diagnosis and therapy. However, the specific function of miR‐9 in hepatocellular carcinoma (HCC) remains unclear, and the mechanisms that underlie HCC are incompletely understood. Here, we report that miR‐9 expression was significantly decreased in clinical tumor tissue samples, as well as in a cohort of HCC cell lines. In addition, it was demonstrated that overexpression of miR‐9 suppressed the proliferative and migratory capacity of HCC cells and impaired cell cycle progression. Furthermore, high mobility group AT‐hook 2 (HMGA2) was verified as a downstream target gene of miR‐9 using a luciferase reporter assay. Quantitative RT‐PCR and western blotting implicated HMGA2 in the miR‐9‐mediated reduction of HCC cell growth. *In vivo*, transfection with miR‐9 mimics down‐regulated the expression of HMGA2, thus leading to a dramatic reduction in tumor growth in a mouse xenograft model. These results suggest that miR‐9 may exert critical antitumor effects on HCC by directly targeting HMGA2, and the miR9/HMGA2 signaling pathway may be of use for the diagnosis and prognosis of patients with HCC.

**Abbreviations**
GAPDH: glyceraldehyde‐3 phosphate dehydrogenase
HCC: hepatocellular carcinoma
HMGA2: high mobility group AT‐hook 2
miRNA: microRNA
NC: negative control
qRT‐PCR: quantitative RT‐PCR
SD: standard deviation

Hepatocellular carcinoma (HCC) is the sixth most prevalent cancer worldwide and is considered one of the main causes of cancer‐related deaths, particularly in China 1, 2. Chronic viral hepatitis B and C infections, alcoholic or nonalcoholic steatohepatitis, aflatoxin intoxication and interactions among these factors have been verified to be involved in the initiation and progression of HCC 3, 4. Notably, only 30%–40% of patients can undergo curative resection, and the prognosis of patients with HCC remains poor because of high rates of cancer metastasis and disease recurrence. Although recent advances in HCC therapy and in functional genomics have led to the development of targeted therapies 5, 6, the pathogenesis and potential molecular targets for prognosis prediction in HCC have yet to be fully characterized.

High mobility group AT‐hook 2 (HMGA2) expression was increased in many human cancers, such as colorectal, breast, ovarian and gastric cancers 7, 8, 9, 10. Because HMGA2 immunopositivity is associated with advanced stage disease and tumor aggressiveness, HMGA2 has emerged as a tumor biomarker 11. Some studies have shown that HMGA2 regulated cell cycle progression, differentiation and cellular senescence, while enhancing or suppressing the expression of several genes 12.

A wide range of studies have demonstrated that microRNAs (miRs or miRNAs) exert an inhibitory effect on tumor growth and may improve the sensitivity of chemotherapy drugs 13, 14. miRNAs are small noncoding RNAs that function as important regulators of human gene expression 15, 16, with >1500 human miRNAs documented in the miRBase database to date that are known to affect a number of diverse cellular processes in embryonic development and disease states 17, 18, 19. In addition, a growing number of studies have indicated that miRNAs are strongly associated with multiple cellular functions, including proliferation, migration, apoptosis, cell cycle progression and angiogenesis, which contribute to tumorigenesis 20, 21. miRNAs, such as miR‐200, miR‐21 and miR‐34b/c, have recently been demonstrated to act as potent regulators during the development and progression of HCC 22, 23, 24. miR‐9 is known to play an important role in tumorigenesis and cancer progression. In brain tumors, miR‐9 is selectively expressed in neural tissues under normal conditions and mediates their development. The expression of miR‐9 has been found to be elevated in the neural tissues of brain tumors when compared with tumors of other histological subtypes, and exhibits a tissue‐specific expression pattern 25, 26. In addition, Talin‐1, which serves a significant role in regulating the transmutation of carcinomas, has been demonstrated to be down‐regulated by miR‐9 in ovarian epithelial cancer 27. However, the role of miR‐9 in HCC remains largely unknown, and its potential downstream targets have not yet been fully defined.

The aim of the present study was to determine the expression of miR‐9 in clinical HCC tissue samples and in various HCC cell lines, to investigate the function of miR‐9 in HCC *in vitro* and *in vivo*, and to identify the underlying downstream targets implicated in HCC.

## Materials and methods

### Human tissue samples

A total of 168 HCC tissue samples and paired normal adjacent liver tissues were separately obtained from patients undergoing surgery from February 2017 to January 2018. All patients provided permission for the collection of their tissue samples. The experiments were approved by the Ethics Committee of the Sichuan Academy of Medical Sciences and Sichuan Provincial People's Hospital (Sichuan, China), and written informed consent was obtained from each enrolled patient. This study also conformed to the standards set by the Declaration of Helsinki. The tissue specimens were stored at −80 °C for subsequent analysis.

### Cell culture

HCC cell lines (Huh‐7, MHCC97H, LM3 and Hep3B) and normal human hepatocytes (THLE‐3) were purchased from the Shanghai Institute of Biochemistry and Cell Biology, Chinese Academy of Sciences (Shanghai, China). The cells were maintained in Dulbecco's modified Eagle's medium supplemented with 10% FBS (Gibco; Thermo Fisher Scientific, Inc., Waltham, MA, USA) in a humidified water‐jacket incubator at 37 °C with 5% CO_2_.

### Quantitative RT‐PCR

Cells were used for *in vitro* experiments only when 90% confluence was reached. Total RNA of HCC tissues, the adjacent normal tissues and cultured cells was extracted according to the instructions of TRIzol and then turned into cDNA according to the instructions of reverse transcription. The target gene and reference gene *GAPDH* (glyceraldehyde‐3 phosphate dehydrogenase) were amplified with cDNA as a template. cDNA was also used to analyze miR‐9 expression using TaqMan assays as previously described 28. Primers are as follows: HMGA2, forward primer 5′‐ACCCAGGGGAAGACCCAAA‐3′, reverse primer 5′‐CCTCTTGGCCGTTTTTCTCCA‐3′; GAPDH, forward primer 5′‐TGTGGGCATCAATGGATTTGG‐3′, reverse primer 5′‐ACACCATGTATTCCGGGTCAAT‐3′; miR‐9: forward primer 5′‐GCGCGTCTTTGGTTATCTAGCTGTATG‐3′, reverse primer 5′‐GCTGTCAACGATACGCTACG‐3′; and *U6* (housekeeping gene): forward primer 5′‐ CGCAAGGATGACACG‐3′, reverse primer 5′‐GAGCAGGCTGGAGAA‐3′.

### Bioinformatic analysis of miR‐9 target genes

The predicted targets and binding sites of miR‐9 were identified using several online programs consisting of databases that use different algorithms, including EIMMO (http://www.mirz.unibas.ch/ElMMo3/) and miRanda‐mirSVR ( http://microRNA.org/).

### Dual‐luciferase assays

In wild‐type (WT) pMIR‐HMGA2‐wt (Genechem, Shanghai, China), with human genomic DNA as a template, PCR was used to amplify the 3′ UTR fragments containing the binding sites of miR‐9. Then the 3′ UTR was cloned into pmirGLO report carrier, which was transformed into the *Escherichia coli* DH5 alpha; random selection of positive PCR was used to identify the recombinant plasmid. In mutant pMIR‐HMGA2‐mut vectors (Mut; Genechem), the mutant 3′ UTR fragment was cloned into the pmirGLO report carrier. Liver cancer cells were plated in 24‐well plates and cotransfected with either WT pMIR‐HMGA2‐wt or mutant pMIR‐HMGA2‐mut vectors, together with miR‐9 mimics (Genechem) or negative control mimics (miR‐NC; Genechem), and the pRL‐TK vector containing the *Renilla* luciferase gene (Promega Corporation, Madison, WI, USA) using Lipofectamine 2000 (catalog no. 11668019; Thermo Fisher Scientific, Inc.). At 48 h after transfection, reporter activity was analyzed using the Dual‐Luciferase Reporter assay system (Promega Corporation) according to the manufacturer's instructions.

### Cell transfection

When cultured liver cancer cells had reached >75% confluence, they were seeded into six‐well plates and cultured for 24 h. Cells were then cotransfected with miR‐NC, miR‐9 mimics, NC‐inhibitor, miR‐9 inhibitor, HMGA2, HMGA2 siRNA, miR‐9 mimics plus HMGA2 or miR‐9 inhibitor plus HMGA2 siRNA using a reagent kit (Invitrogen; Thermo Fisher Scientific, Inc., USA) according to the manufacturer's protocol. The following sequences were used: miR‐9, 5′‐UCAUACAGCUAGAUAACCAAAGA‐3′; miR‐NC, 5′‐UCACAGUGAACCGGUCUCUUU‐3′; miR‐9 inhibitor, 5′‐UCAUACAGCUAGAUAACCAAAGA‐3′; and NC‐inhibitor, 5′‐CAGUACUUUUGUGUAGUACAA‐3′.

### Western blot analysis

Transfected liver cancer cells were collected, and total proteins of HCC tissues, the adjacent normal tissues and cultured cells were separated according to our protocols. In brief, radioimmunoprecipitation assay buffer was used to crack the collected cells; after centrifugation (13 400 ***g***, 30 min, 4 °C), the supernatant was collected into a 1.5‐mL EP tube that was stored at −80 °C in a refrigerator. Protein concentration was detected according to bicinchoninic acid kit instructions. Western blot analysis was performed as previously described 29. In brief, immunodetection of HMGA2 was achieved using the rabbit polyclonal anti‐HMGA2 serum (dilution, 1 : 1000; Sigma, Merck, KGaA, Darmstadt, Germany). GAPDH (catalog no. G5262; Sigma, Los Angeles, CA, USA) was used as an internal control. Horseradish peroxidase antibody (1 : 5000, ab6728; Abcam, Cambridge, MA, USA) was incubated and added with enhanced chemiluminescence chromogenic liquid for color imaging.

### Cell proliferation, invasion and migration assays

Transfected liver cancer cells were seeded in 96‐well plates and assayed for proliferation at 48 h using a Cell Counting Kit‐8 kit (Dojindo Molecular Technologies, Inc., Kumamoto, Japan) according to the manufacturer's protocol. Cell proliferation was analyzed by measuring the absorbance at 450 nm using a microplate reader. Liver cancer cells (5 × 10^5^) were seeded into six‐well plates and cultured under standard conditions. When the cell density reached confluence, a ‘wound’ was generated by scraping the cell monolayer with a 200‐μL pipette tip. Cell migration was determined by measuring the movement of cells into the scraped area. The process of wound closure was monitored and photographed at 12 h after wound generation using a microscope. For the invasion experiment, the low growth factor matrix was mixed with serum‐free medium (1 : 4) and 60 μL mixed matrix was spread on the Transwell chamber. Then, the small chamber was placed in the 24‐well plate, which was placed into the constant temperature incubator for 4–6 h. The liver cancer cells were digested after transfection for 24 h, a total of 8 × 10^4^ cells were placed in the Transwell chamber, in which the lower chamber was the normal culture medium containing 10% FBS, and after that, the Transwell chamber was placed in the constant temperature incubator for 48 h. The small chamber was taken out, the cells were fixed with 4% paraformaldehyde for 10 min, and crystal violet was used to stain cells for 10 min. The positive cells were counted and photos were taken. For the migration experiment, the Transwell transfer film was flattened out on the 24‐well plate containing the Dulbecco's modified Eagle's medium with 10% serum. Resuspended cells were added and cultured in the well for 24 h. The cells passing through the Transwell membrane were then immobilized in 4% paraformaldehyde and photographed.

### Flow cytometry

Transfected liver cancer cells were suspended, collected and then washed twice with saline before they were fixed with 70% ethyl alcohol. Subsequently, 5 mL Annexin V/FITC and 10 mL propidium iodide [from the Annexin V/PI apoptosis kit; Hangzhou MultiSciences (Lianke) Biotech, Co., Ltd., Hangzhou, China] were added to each sample for staining at 37 °C in the dark. The liver cancer cell cycle was detected using the BD FACSCalibur (BD Biosciences, Franklin Lakes, NJ, USA), and data were analyzed using flowjo software (Tree Star, Inc. Ashland, OR, USA).

### Mouse xenograft model

All procedures were performed in accordance with national (D.L.n.26, March 4, 2014) and international laws and policies (directive 2010/63/EU), and were approved by the Animal Experimental Ethics Committee, Sichuan Academy of Medical Sciences and Sichuan Provincial People's Hospital. HCC‐bearing nude mice (male; aged 4–5 weeks; weight, 15–17 g) were raised and maintained using the same methods described previously 30, 31. To generate the mouse model of HCC, we first transfected liver cancer cells with 150 nm miR‐9 mimics or miR‐NC using Nucleofector II (Amaxa Biosystems; Lonza Group, Ltd., Basel, Switzerland). After transfection, the cells were allowed to recover in fresh medium by incubating at 37 °C for 24 h. Subsequently, the cells were collected and washed with ice‐cold PBS three times before they were resuspended in PBS at a density of 5 × 10^6 ^cells·mL^−1^. Nude mice were divided into two groups (*n* = 8) and subcutaneously injected with 5 × 10^5^ cells (100 μL) into the left flank. Tumor growth was measured every 3 days, and tumor volume (*V*) was monitored by measuring the length (*L*) and width (*W*) with calipers, and calculated using the following formula: *V* = (*L *× *W* ^2^) × 0.5. Euthanasia was carried out by cervical dislocation after rendering mice unconscious with CO_2_. The tumors were excised and weighed on day 22, before being preserved in 4% paraformaldehyde at 4 °C for immunohistochemical analysis. All of the procedures were performed in accordance with national (D.L.n.26, March 4, 2014) and international laws and policies (directive 2010/63/EU); they also were approved by the Animal Experimental Ethics Committee, Sichuan Academy of Medical Sciences and Sichuan Provincial People's Hospital.

### Immunohistochemical analysis

The expression of HMGA2 and Ki‐67 in tumor sections was measured using an immunohistochemistry assay as described previously 32. Rabbit polyclonal anti‐HMGA2 (dilution, 1 : 200; Sigma, Merck, KGaA) and anti‐Ki‐67 (dilution, 1 : 200; CST, Cambridge, MA, USA) sera were used.

### Statistical analysis

All quantitative data for statistical analyses were derived from at least three independent experiments. Data are presented as the mean ± standard deviation (SD). Student's *t*‐test was used to compare the mean values between the two groups; ANOVA test (Bonferroni as the post hoc test) was used to compare the mean among three or more groups. A *P*‐value <0.05 was considered to indicate a statistically significant difference.

## Results

### Expression of miR‐9 and HMGA2 in HCC cell lines and tissue samples

**Figure 1:**
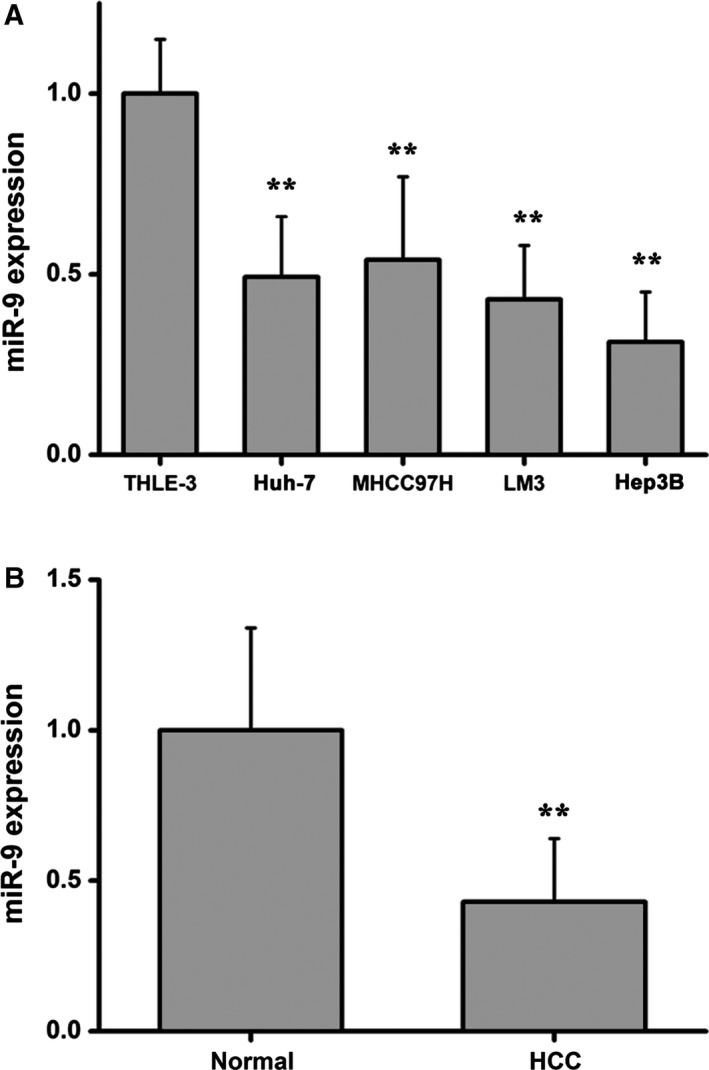
miR‐9 is differentially expressed in HCC cells and in clinical cancer specimens. (A) miR‐9 expression in HCC cell lines (Huh‐7, MHCC97H, LM3 and Hep3B) and normal human hepatocytes (THLE‐3). Data were expressed as the mean ± SD, one‐way ANOVA. (B) miR‐9 expression in HCC and paired adjacent liver tissues. Data were expressed as the mean ± SD, Student's *t*‐test. *n* = 6. **P < 0.01.

**Figure 2:**
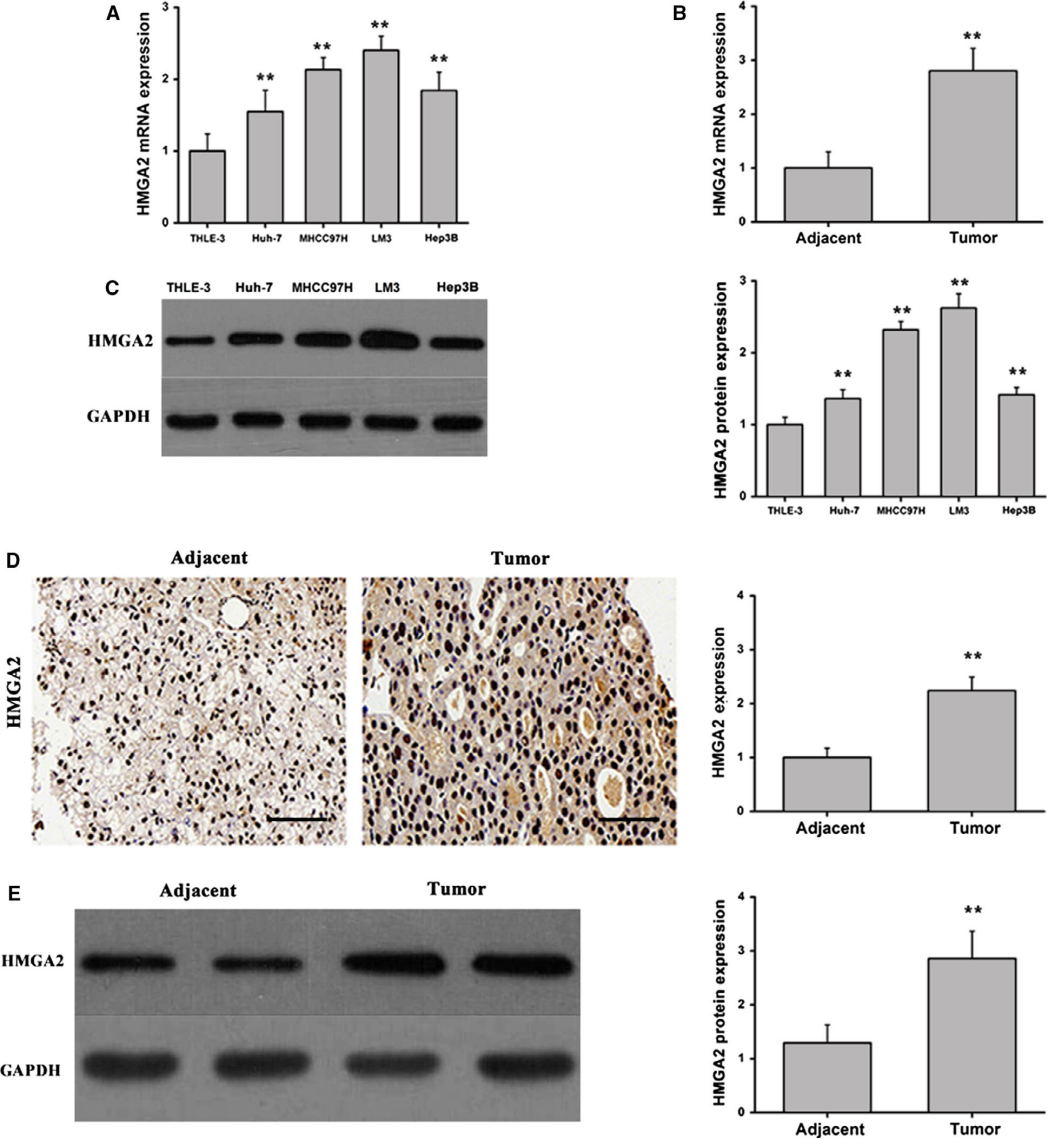
HMGA2 is differentially expressed in HCC cells and clinical cancer specimens. (A) HMGA2 mRNA expression in HCC cell lines (Huh‐7, MHCC97H, LM3 and Hep3B) and normal human hepatocytes (THLE‐3). Data were expressed as the mean ± SD, one‐way ANOVA. (B) HMGA2 expression in HCC tissues and paired adjacent liver tissues. Data were expressed as the mean ± SD, Student's *t*‐test. (C) HMGA2 protein expression was measured by western blot in HCC cell lines (Huh‐7, MHCC97H, LM3 and Hep3B) and normal human hepatocytes (THLE‐3). Data were expressed as the mean ± SD, one‐way ANOVA. (D) HMGA2 protein expression was measured by immunohistochemical staining and quantitated (scale bars: 50 μm). Data were expressed as the mean ± SD, Student's *t*‐test. (E) The western blot and quantification of HMGA2 expression in adjacent and tumor tissue. Data were expressed as the mean ± SD, Student's *t*‐test. *n* = 6. **P < 0.01.

To investigate the role of miR‐9 in HCC, we employed quantitative RT‐PCR (qRT‐PCR) to detect miR‐9 expression in HCC cell lines (Huh‐7, MHCC97H, LM3 and Hep3B) and the normal hepatic cell line, THLE‐3. As shown in Fig. 1A, miR‐9 exhibited reduced expression in the four HCC cell lines when compared with normal hepatocytes. In addition, miR‐9 expression was significantly down‐regulated in HCC biopsy tissues when compared with adjacent normal tissues (Fig. 1B). These findings indicate that miR‐9 may be involved in the development and progression of HCC. In contrast with miR‐9 expression, HMGA2, a potential target of miR‐9, was highly expressed in HCC cell lines (Fig. 2A) and in clinical tumor samples (Fig. 2B) when compared with normal controls. In line with these observations, the quantitative analysis of HMGA2 by immunohistochemical staining demonstrated that HMGA2 was highly expressed in clinical tumor samples when compared with paired adjacent normal tissues (Fig. 2D). Meanwhile, the quantitative analysis of HMGA2 protein by western blot showed the same tendency (Fig. 2C,E). These results indicate that miR‐9 expression is inversely correlated with HMGA2 expression.

### miR‐9 regulates HMGA2 expression via targeting the 3′ UTR of HMGA2

**Figure 3:**
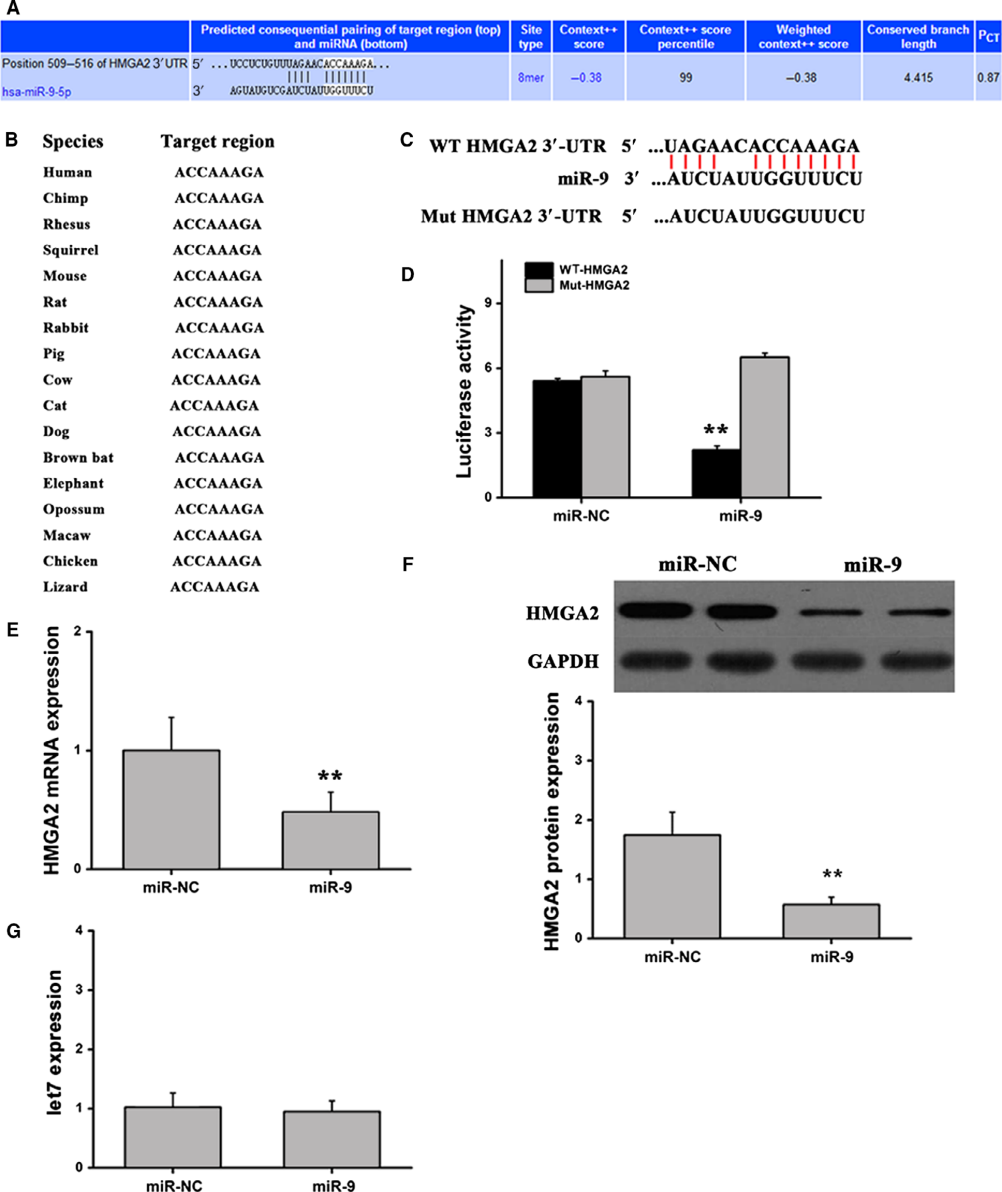
miR‐9 regulates HMGA2 expression via binding to the 3′UTR of HMGA2 *in vitro*. (A) The predicted target sequence of miR‐9 in the 3′ UTR of HMGA2. (B) Highly conserved sequences in different species. (C) WT or mutant (Mut) miR‐9 target sequences of the 3′ UTR of HMGA2. (D) Luciferase activity of cells cotransfected with the WT or Mut HMGA2 3′UTR reporter genes along with miR‐9 mimics or control. (E) Quantification of HMGA2 at the mRNA level by qRT‐PCR. (F) HMGA2 proteins were determined by western blotting. (G) The expression of let7 was determined by qRT‐PCR. *n* = 6. **P < 0.01. Data were expressed as the mean ± SD, Student's *t*‐test.

To investigate the potential targets of miR‐9, using accessible databases, we screened the 3′ UTR of HMGA2 for any predicted miR‐9‐binding sequences. Interestingly, a conserved binding site for miR‐9 was identified in the 3′ UTR of HMGA2 (Fig. 3A,B). It has been reported that high HMGA expression levels are strongly associated with the progression, metastasis and poor prognosis of specific human cancers and present a robust molecular biomarker for diagnosis 33, 34, 35. To determine whether miR‐9 binds to the 3′ UTR of HMGA2, we mutated the miR‐9 binding site in the pMIR‐HMGA2‐wt luciferase reporter to generate a mutant pMIR‐HMGA2‐mut reporter (Fig. 3C). The pMIR‐HMGA2‐wt or pMIR‐HMGA2‐mut reporter was cotransfected into liver cancer cells together with miR‐9 or miR‐NC mimics, and luciferase activity was measured. Transfection with miR‐9 mimics demonstrated that the luciferase activity of pMIR‐HMGA2‐wt was significantly decreased when compared with the control. However, transfection with miR‐9 mimics did not affect the luciferase activity of the pMIR‐HMGA2‐mut reporter gene, suggesting that miR‐9 can target the 3′ UTR of HMGA2 (Fig. 3D). In addition, HMGA2 expression in cells transfected with miR‐9 mimics was determined to confirm whether miR‐9 can successfully regulate HMGA2 expression. As expected, miR‐9 significantly reduced HMGA2 expression at the mRNA and protein levels (Fig. 3E,F). HMGA2 is a target of let7 family miRNAs 36. We detected whether miR‐9 regulated HMGA2 expression via effecting let7, and qRT‐PCR showed that overexpression of miR‐9 did not regulate the level of let7 (Fig. 3G). These results reveal that miR‐9 may inhibit the expression of HMGA2 in HCC cells via directly binding to the 3′ UTR of HMGA2.

### Effects of miR‐9 on the proliferation, invasion, migration and cell cycle progression of HCC cells

**Figure 4:**
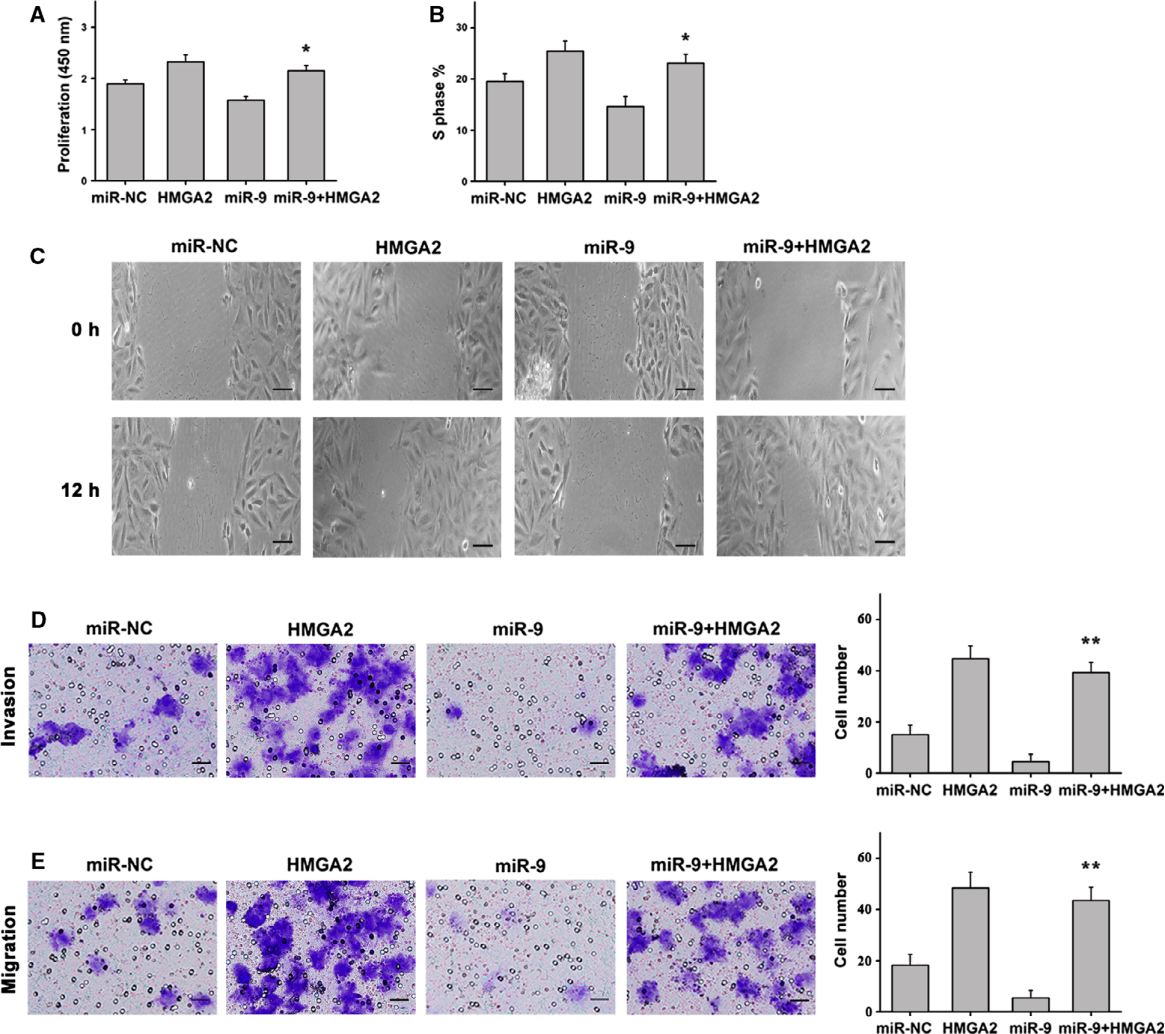
Effect of miR‐9 in HCC cells. (A) Cell proliferation was reduced in HCC cells transfected with miR‐9 mimics when compared with the controls. The opposite results were observed in cells treated with miR‐9 mimics plus HMGA2. (B) Cells transfected with miR‐9 mimics showed a reduction in the number of cells entering S phase, whereas treatment with miR‐9 mimics plus HMGA2 exerted the opposite effect. (C) Representative images of migrated cells transfected with miR‐9 mimics, control mimics, HMGA2 or miR‐9 mimics plus HMGA2 (scale bars: 25 μm). (D) The invasion of cells transfected with miR‐9 mimics, control mimics, HMGA2 or miR‐9 mimics plus HMGA2 (scale bars: 50 μm). (E) The migration of cells transfected with miR‐9 mimics, control mimics, HMGA2 or miR‐9 mimics plus HMGA2 (scale bars: 50 μm). *n* = 6. *P < 0.05; **P < 0.01. Data were expressed as the mean ± SD, one‐way ANOVA.

**Figure 5:**
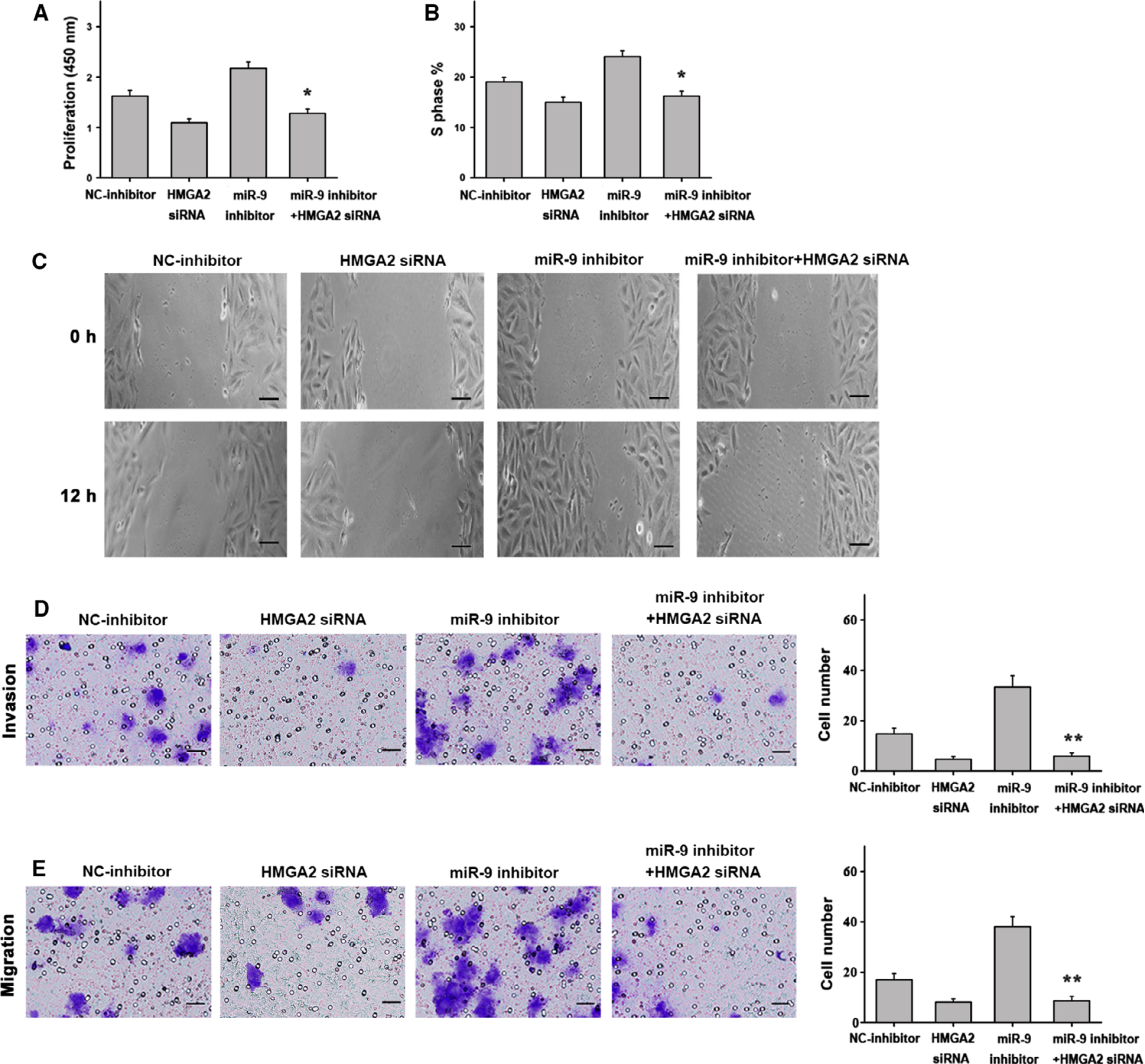
Effect of miR‐9 inhibitor in HCC cells. (A) Cell proliferation was increased in HCC cells transfected with miR‐9 inhibitor when compared with the controls. The opposite results were observed in cells treated with the miR‐9 inhibitor plus HMGA2 siRNA. (B) Cells transfected with the miR‐9 inhibitor showed an increase in the number of cells entering S phase, whereas treatment with the miR‐9 inhibitor plus HMGA2 siRNA exerted the opposite effect. (C) Representative images of migrated cells transfected with miR‐9 inhibitor, NC‐inhibitor, HMGA2 siRNA or miR‐9 inhibitor plus HMGA2 siRNA (scale bars: 25 μm). (D) The invasion of cells transfected with the miR‐9 inhibitor, NC‐inhibitor, HMGA2 siRNA or miR‐9 inhibitor plus HMGA2 siRNA (scale bars: 50 μm). (E) The migration of cells transfected with the miR‐9 inhibitor, NC‐inhibitor, HMGA2 siRNA or miR‐9 inhibitor plus HMGA2 siRNA (scale bars: 50 μm). *n* = 6. *P < 0.05; **P < 0.01. Data were expressed as the mean ± SD, one‐way ANOVA.

Considering the observed reduction in miR‐9 expression in different types of HCC cell lines and in clinical HCC tumor tissues, the functional relevance of miR‐9 in the hepatic cancer phenotype was investigated further. To assess the impact of miR‐9 on cell growth, we transfected liver cancer cells with miR‐NC, miR‐9 mimics, HMGA2 or miR‐9 mimics plus HMGA2. Using the Cell Counting Kit‐8 assay, we found that cells transfected with miR‐9 mimics exhibited reduced cell proliferation when compared with the control, which was rescued by transfection of miR‐9 mimics and HMGA2 (Fig. 4A). In addition, flow cytometry analysis showed that cell cycle progression from the G0/G1 to the S phase was significantly suppressed in cells transfected with miR‐9 mimics. Conversely, treatment with miR‐9 mimics and HMGA2 induced S‐phase arrest in HCC cells (Fig. 4B). Wound closure assays were performed to assess cell migration, and the results indicated that transfection with miR‐9 mimics significantly attenuated the migration of liver cancer cells. In contrast, liver cancer cells transfected with miR‐9 mimics plus HMGA2 exhibited an increase in migration capacity (Fig. 4C). Transwell assays showed that overexpression of miR‐9 alone could inhibit the migration and invasion of liver cancer cells. When miR‐9 and HMGA2 were overexpressed at the same time, the inhibition effect of miR‐9 on the migration and invasion of liver cancer cells disappeared, and the migration and invasion behavior of the cells was aggravated (Fig. 4D,E). Meanwhile, liver cancer cells transfected with NC‐inhibitor, miR‐9 inhibitor, HMGA2 siRNA or miR‐9 inhibitor plus HMGA2 siRNA. Liver cancer cells transfected with the miR‐9 inhibitor exhibited an increase in proliferation, S‐phase cell fraction, cell migration and invasion capacity of HCC cells, whereas down‐regulation of miR‐9 expression and interference with HMGA2 exhibited an inhibition in proliferation, S‐phase cell fraction and migration capacity of HCC cells (Fig. 5A–E). These results suggest that miR‐9 may play a crucial role in HCC cells by reducing their proliferative and migratory capacity and by impairing cell cycle progression. In contrast, ectopic expression of HMGA2 may reverse the antitumor effects of miR‐9.

### miR‐9 inhibits tumor growth in mice

**Figure 6:**
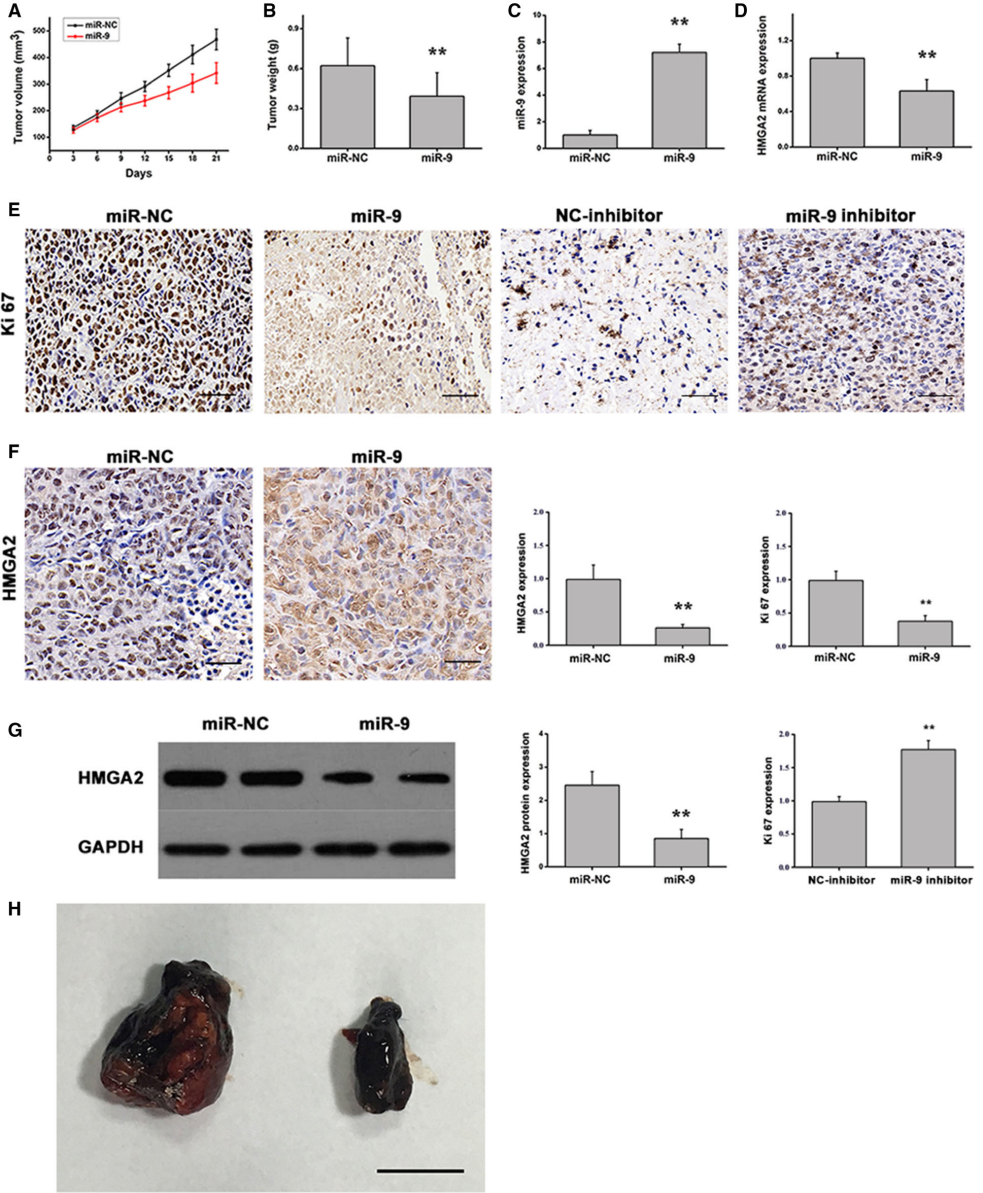
miR‐9 inhibits tumor growth *in vivo*. (A) Tumor volume growth curves. (B) Tumor weight. (C) miR‐9 expression. (D) HMGA2 mRNA expression. (E) Ki‐67 protein expression was measured by immunohistochemical staining (scale bars: 50 μm). (F) HMGA2 protein expression was measured by immunohistochemical staining (scale bars: 50 μm). (G) HMGA2 protein expression was measured by western blot. (H) Tumor images (scale bar: 1 cm). *n* = 8. **P < 0.01. Data were expressed as the mean ± SD, Student's *t*‐test.

Liver cancer cells were used to generate a mouse HCC xenograft tumor model for further *in vivo* experiments. Cells transfected with miR‐9 or miR‐NC mimics were injected subcutaneously into the left flank of nude mice, and tumor growth was monitored for 21 days. At this point, the tumors were excised, weighed and photographed. Treatment with miR‐9 mimics significantly reduced the tumor volume and weight when compared with the controls (Fig. 6A,B,H). To investigate the mechanisms by which miR‐9 suppressed liver cancer tumor cell growth, we detected the expression of miR‐9 and HMGA2 in mouse tumors by qRT‐PCR. The results demonstrated that miR‐9 was highly expressed in tumor tissues transfected with miR‐9 mimics (Fig. 6C), whereas the mRNA and protein levels of HMGA2 were also reduced by qRT‐PCR, immunohistochemistry and western blot (Fig. 6D,F,G). In addition, immunohistochemical staining analysis of the Ki‐67 marker of cell proliferation in mouse tumor tissue samples revealed that transfection with miR‐9 mimics decreased the expression of Ki‐67, whereas transfection with the miR‐9 inhibitor promoted the expression of Ki‐67, compared with controls (Fig. 6E). Taken together, these observations suggest that miR‐9 may suppress tumor growth by inhibiting HMGA2 expression.

## Discussion

miRNAs are considered critical regulators of numerous cellular processes, including cell proliferation, migration, apoptosis, differentiation, cell cycle progression and carcinogenesis 37, 38, 39. In addition, accumulating evidence suggests that miRNAs are closely associated with the initiation and progression of malignant tumors 40, 41. Although the present study is not the first to identify the functional role of miR‐9 in HCC, a number of novel findings have been presented. To the best of our knowledge, this is the first study to demonstrate that miR‐9 expression is closely correlated with hepatic tumor differentiation. In addition, miR‐9 overexpression exerted an inhibitory effect on the proliferation, migration and cell cycle progression of HCC cells. Furthermore, HMGA2 was identified as a potential target of miR‐9, and the miR‐9/HMGA2 signaling pathway may be involved in HCC. It could be hypothesized that miR‐9 may display antitumor activity by directly binding to HMGA2, which may contribute to its use as a diagnostic and/or prognostic marker for patients with HCC.

Currently, a number of specific miRNAs have been verified to be aberrantly expressed in liver cancers characterized by poor prognosis. Coulouarn *et al*. 42 reported that miR‐122 is a biomarker of hepatocyte‐specific differentiation and plays a causal role in regulating cell migration and invasion in HCC. Additional studies have demonstrated that miRNAs, including miR‐21, miR‐29a/b, miR‐26a, miR‐101 and miR‐375, are associated with the pathogenesis of HCC by activating intricate signaling cascades 23, 43, 44, 45, 46. It has been reported that miR‐9 expression was decreased in a variety of cancers and was associated with tumor invasion and progression 47, 48, 49; however, the role of miR‐9 in HCC pathobiology is not well understood. In a recent study, our research group demonstrated a link between miR‐9 expression and the extent of tissue differentiation of HCC‐derived cells. By analyzing the expression of miR‐9 in primary HCC and adjacent tissues, as well as in a cohort of HCC‐derived cells, miR‐9 expression was observed to be significantly down‐regulated in primary hepatic tumor tissues and in different strains of HCC cells, suggesting that miR‐9 may be strongly associated with hepatocyte differentiation and affect hepatic metabolic homeostasis. Therefore, the role of miR‐9 in HCC was investigated further in the present study.

Several previous studies have demonstrated that miR‐9 is selectively expressed in neural tissues 26, and its expression levels are enriched in brain tumors 27 and in clinical breast cancer samples 50. In addition, miR‐9 has been found to play an oncogenic role in HCC by inducing cell growth and invasiveness 51. To improve our understanding of the function of miR‐9 in HCC, we performed a series of experiments investigating the impact of miR‐9 on the hepatic cancer phenotype in the present study. The effect of miR‐9 on HCC cell growth was analyzed *in vitro* and *in vivo*. The results demonstrated that overexpression of miR‐9 significantly attenuated cell proliferation and migration, and impaired S‐phase arrest in HCC cells. However, down‐regulation of mir‐9 expression had the opposite results. Consistent with the *in vitro* results, miR‐9 exerted antitumor effects by reducing the tumor weight and size in an *in vivo* mouse xenograft model. Taken together, these results suggest that miR‐9 may act as a tumor suppressor by regulating cell growth, migration and cell cycle progression in HCC, which is consistent with the results of a recent study 52.

To further understand the mechanisms underlying the observed ability of miR‐9 to regulate HCC cell proliferation, migration and cell cycle progression, in the present study we used bioinformatic analysis to identify HMGA2 as a potential target gene of miR‐9. HMGA2 is an abundant, nonhistone chromatin architectural factor that has been implicated in multiple biological processes, including cell growth and differentiation 53. In addition, HMGA2 is highly expressed in the developing embryo and displays down‐regulated expression during differentiation 54, 55. Notably, previous studies have provided evidence showing that overexpression of HMGA at the protein level in all tissues of transgenic mice was associated with the development of lymphomas and other tumor types 56, 57. Furthermore, HMGA was found to be strongly associated with metastasis and poor prognosis in some human cancers 58, 59. However, the mechanisms underlying the role of HMGA2 in HCC remain unclear. Notably, an evolutionarily conserved binding site for miR‐9 in the 3′ UTR of HMGA2 was identified using a luciferase reporter gene experiment in the present study. In addition, transfection with miR‐9 mimics strongly down‐regulated HMGA2 expression both at the mRNA and the protein levels in HCC cells, indicating that miR‐9 may play a key role in HCC, particularly when combined with the reduced expression of HMGA2.

A close association between increased HMGA expression and the progression, metastasis and poor prognosis of multiple human cancers has been observed, and HMGA may also serve as a molecular biomarker for the diagnosis of certain cancers 33, 34, 35. Watanabe *et al*. 60 demonstrated that HMGA2 is involved in maintaining epithelial‐to‐mesenchymal transition in pancreatic cancer, which may represent a promising therapeutic strategy. In addition, the acquisition of antitumor properties was observed when single knockdown of HMGA2 induced long‐term growth inhibition in pancreatic cancer. Furthermore, HMGA2 was found to affect the cyclin A gene and promote cell growth 61. Thus, HMGA2 appears to be a determinant of cell invasiveness and metastasis. In the present study, HMGA2 was found to be highly expressed in a cohort of HCC cell lines and in primary hepatic tumor tissues using qRT‐PCR analysis, suggesting that HMGA2 may contribute to HCC progression. A cotransfection HCC cell model was established to further understand the relationship between miR‐9 and HMGA2 and its role in the hepatic cancer phenotype. The results indicated that overexpression of miR‐9 exerted an inhibitory effect on the proliferation, migration and cell cycle progression of HCC cells, whereas HMGA2 significantly reversed these effects when transiently cotransfected with miR‐9 mimics. In addition, we also found that down‐regulation of miR‐9 expression promoted malignant biological properties of HCC cells, whereas miR‐9 inhibitor plus HMGA2 siRNA cotransfection reversed this promoting effect. Furthermore, an *in vivo* model was established to further investigate the underlying association between miR‐9 and HMGA2. Treatment with miR‐9 mimics inhibited tumor growth, potentially via down‐regulating HMGA2 expression. Taken together, these findings confirm that HMGA2 may be a functional target of miR‐9, and activation of the miR‐9/HMGA signaling pathway may be a potential mechanism that may be exploited for the prevention and/or treatment of HCC.

## Conclusions

In conclusion, the results of the present study provide a rationale for the potential use of miR‐9 as a novel diagnostic and therapeutic marker that is involved in the development and progression of HCC. miR‐9 may play a crucial role in the proliferation, migration and cell cycle progression of HCC cells *in vitro* and in HCC tumor growth *in vivo* via targeting HMGA2. Ectopic HMGA2 expression may reverse the antitumor effects of miR‐9, suggesting that the miR‐9/MHGA2 signaling pathway may present a promising therapeutic target for patients with HCC.

## Conflict of interest

The authors declare no conflict of interest.

## Author contributions

XX, HZ and GW conceived and designed the project. XX, HZ, LL and XW acquired the data. XX, HZ, LL and GW analyzed and interpreted the data. All authors wrote the paper.
